# Hypogonadotropic Hypogonadism in Diabesity: Pathogenic Factors and Therapeutic Implications

**DOI:** 10.1089/andro.2022.0019

**Published:** 2022-12-28

**Authors:** Paresh Dandona, Sandeep Dhindsa, Husam Ghanim

**Affiliations:** ^1^ Division of Endocrinology, Diabetes and Metabolism, State University of New York at Buffalo, Williamsville, New York, USA.; ^2^ Division of Endocrinology, Diabetes and Metabolism, Saint Louis University, St. Louis, Missouri, USA.

Hypogonadotropic hypogonadism (HH) was found to occur in 33% male patients with type 2 diabetes.^1^ These patients had low total and free testosterone concentrations with inappropriately low or normal luteinizing hormone (LH) and follicle-stimulating hormone (FSH) concentrations. Since the occurrence of hypogonadism was not related to either HbA1c or the duration of diabetes but to body mass index (BMI), a study in nondiabetic obese patients was carried out, which revealed a prevalence of HH in 25%.^2^ Thus, this is the commonest cause of hypogonadism in the community.

These observations have added ∼18 million hypogonadal patients in the United States alone, based on the prevalence of type 2 diabetes and obesity. In a study comparing type 1 and type 2 diabetic patients, it was shown that the occurrence of HH was confined to type 2 diabetes.^3^ Since these observations were made in middle aged to older populations, a study in obese young males between 14 and 20 years of age was conducted.^4^ These patients were found to have similar prevalence of HH as reflected in the total and free testosterone, LH, and FSH concentrations.

In these patients and older patients, testosterone concentrations were found to have an inverse relationship with BMI. Bariatric surgery with weight loss has been shown to raise testosterone concentrations. In the younger population with morbid obesity, the prevalence of HH was 75%.^5^ After bariatric surgery, testosterone levels normalized in the majority after 2 years and were maintained in those who maintained their weight loss but diminished again if they regained weight. Thus, body weight is a major determinant of plasma testosterone concentrations.

It has also been demonstrated that diabetic patients with HH have increased insulin resistance when compared with those without.^6^ Testosterone patients restore insulin sensitivity as measured by Homeostatic Model Assessment of Insulin Resistance or euglycemic hyperinsulinemic clamps, while simultaneously reducing adiposity and increasing muscle mass. In HH patients, four genes in the insulin signaling pathway, insulin receptor β-subunit, IRS-1, Akt-2, and GLUT-4, had diminished expression. All four genes had normalized expression after testosterone replacement.

Thus, testosterone is an insulin sensitizer.^7^ In addition, testosterone also increases the expression of activated protein kinase that induces an increase in glucose uptake independently of insulin signal transduction.^8^ Consistent with these observations, testosterone has been shown to prevent the development of diabetes in prediabetic patients, reverse prediabetes into a state of normality, and to reverse diabetes in 33% of patients with diabetes and HH for an 8-year period.^9,10^

In addition to the mentioned effects, testosterone also increased the expression of the androgen receptor, the estrogen receptor, and aromatase, all of which are diminished in HH patients.^11^ Furthermore, testosterone also exerts anti-inflammatory effects that may be relevant to the inhibition of atherosclerosis and the control/prevention of infections.^6^ In this context, it is relevant that a very large retrospective study has shown that hypogonadal males have markedly increased cardiovascular mortality, whereas those hypogonadal patients whose testosterone had been replaced appropriately did not have such an increase.^12^

Most recently, hypogonadal males have been shown to have two times higher incidence of hospitalization after COVID-19 infection, as compared with men with normal testosterone concentrations.^13^ This was independent of other known risk factors for hospitalization, such as age, comorbidities, immunosuppression, or body weight. Hypogonadal men with appropriate testosterone replacement did not experience an increase in these hospitalizations. Clearly, therefore, testosterone also has an anti-inflammatory effect that is clinically relevant.

The most recent advance in HH and testosterone replacement was when obese males with either impaired glucose tolerance or early diabetes with testosterone concentrations <400 ng/dL were treated with depot testosterone 1000 mg every 3 months in addition to lifestyle change.^14^ The control arm was subjected to lifestyle change only with placebo injections. It was observed that plasma glucose concentrations fell, and diabetes was reversed in 21.1% of patients on testosterone as compared with 12.4% on lifestyle alone.

There was a decrease in 2 h glucose by 31 mg/dL in the testosterone arm and 17 mg/dL in the control arm. Among the prediabetic patients, 7.6% patients progressed to diabetes in the testosterone arm, whereas 14.9% progressed to diabetes in the control arm. Among the early type 2 diabetic patients, there was a reversal of diabetes in 45.2% in the testosterone arm and only 32.1% in the control arm. In addition, there was a significantly greater reduction in waist circumference, total, and abdominal adiposity in the testosterone arm when compared with the control arm.

Furthermore, there was an increase in total and arm muscle mass and the strength of the muscles in the nondominant hand. This large prospectively randomized study confirmed the previous observations in patients who had low normal rather than subnormal testosterone concentrations,^14^ as already discussed.^6,7^ An Austrian study has also recently confirmed a relationship between testosterone and insulin sensitivity and related metabolic indices in prediabetic males.^15^ Clearly, therefore, HH predisposes individuals to an insulin-resistant state and testosterone restores insulin sensitivity and can potentially reverse type 2 diabetes and prediabetes.^6,15–17^

In conclusion, testosterone needs to be measured in all type 2 diabetes and obese patients, and if the concentrations are low, testosterone replacement has to be considered not only for its effects on sexual function but also for its metabolic effects that are profound and widespread.

## Abbreviations Used

BMI: body mass index
FSH: follicle-stimulating hormone
HH: hypogonadotropic hypogonadism
LH: luteinizing hormone
